# Identification of cattle, buffaloes and rodents as reservoir animals of *Leptospira* in the District of Gampaha, Sri Lanka

**DOI:** 10.1186/s13104-017-2457-4

**Published:** 2017-03-23

**Authors:** D. T. H. Denipitiya, N. V. Chandrasekharan, W. Abeyewickreme, R. A. Hartskeerl, M. D. Hapugoda

**Affiliations:** 1 Molecular Medicine Unit, Faculty of Medicine, University of Kelaniya, P.O. Box 06, Ragama, Sri Lanka; 2 Department of Chemistry, Faculty of Science, University of Colombo, Colombo 03, Sri Lanka; 3 WHO/FAO/OIE Collaborating Centre on Leptospirosis, KIT Biomedical Research, Meibergdreef 39, Amsterdam, Netherlands

**Keywords:** Leptospirosis, Reservoir animals, Real-time PCR, Microscopic agglutination test (MAT)

## Abstract

**Background:**

Leptospirosis is an important emerging infectious disease in Sri Lanka. Rats are the most important reservoir of *Leptospira* but domestic and wild mammals may also act as important maintenance or accidental hosts. In Sri Lanka, knowledge of reservoir animals of leptospires is poor. The objective of this study was to identify potential reservoir animals of *Leptospira* in the District of Gampaha, Sri Lanka.

**Findings:**

Blood and kidney samples were collected from 38 rodents and mid-stream urine samples were randomly collected from 45 cattle and five buffaloes in the District of Gampaha. Kidney and urine samples were tested by real-time polymerase chain reaction (PCR) and serum samples were tested by the microscopic agglutination test (MAT). Of the 38 rodent kidney samples, 11% (4/38) were positive by real-time PCR. The prevalence of leptospiral carriage was 11% (3/26) and 8% (1/12) in female and male rodents, respectively. Three rodent serum samples were positive by MAT. Of the 50 cattle/buffalo urine samples tested, 10% (5/50) were positive by real-time PCR. The prevalence of leptospiral carriage was 9% (4/45) and 20% (1/5) in cattle and buffaloes, respectively.

**Conclusion:**

Results of PCR and MAT showed that *Leptospira* were present in a significant proportion of the rodents and farm animals tested in this study and suggest that these (semi-) domestic animals form an infection reservoir for *Leptospira.* Therefore, there is a potential zoonotic risk to public health, most notably to farmers in this area.

## Background

Leptospirosis is a globally important zoonotic disease which affects humans and animals in countries with humid, tropical and sub-tropical climates [1]. Local annual incidences range from 0.10 to 975 cases per 100,000 in the population [2]. The disease is caused by infection with pathogenic spirochetes of the genus *Leptospira* [3]. The first leptospirosis case in Sri Lanka was reported in 1959 [4]. Since then, more than 1000 cases of leptospirosis annually, including several major outbreaks, have been reported [5]. The disease is distributed throughout the country but during the last two decades the highest numbers of leptospirosis cases have been reported from the Districts of Colombo, Gampaha, Kurunegala, Matale and Kegalle [6]. The levels of laboratory diagnosis and involvement in awareness programs on leptospirosis in the country are poor and activities generally confined to periods of disease outbreak.

The major route of infection is by indirect environmental contact with leptospires excreted in the urine of reservoir hosts [1, 7, 8], so the presence of reservoir animals is an important risk factor for human leptospirosis cases. Rats are notorious reservoir hosts of leptospires but domestic and wild animals may be transient or persistent shedders of leptospires [9–11]. Unfortunately, people living in Sri Lanka have a poor level of knowledge around potential risk factors for leptospirosis [12]. A few studies on leptospirosis have been carried out in the country but knowledge of reservoir animals of pathogenic *Leptospira* species in the highly endemic District of Gampaha is lacking. In recent years, the district has reported more than 400 cases of leptospirosis annually [13], and so the goal of this study was to determine whether cattle, buffaloes or rodents are potential reservoir animals of leptospires in Gampaha, Sri Lanka.

## Methods

### Study area

**Fig. 1:**
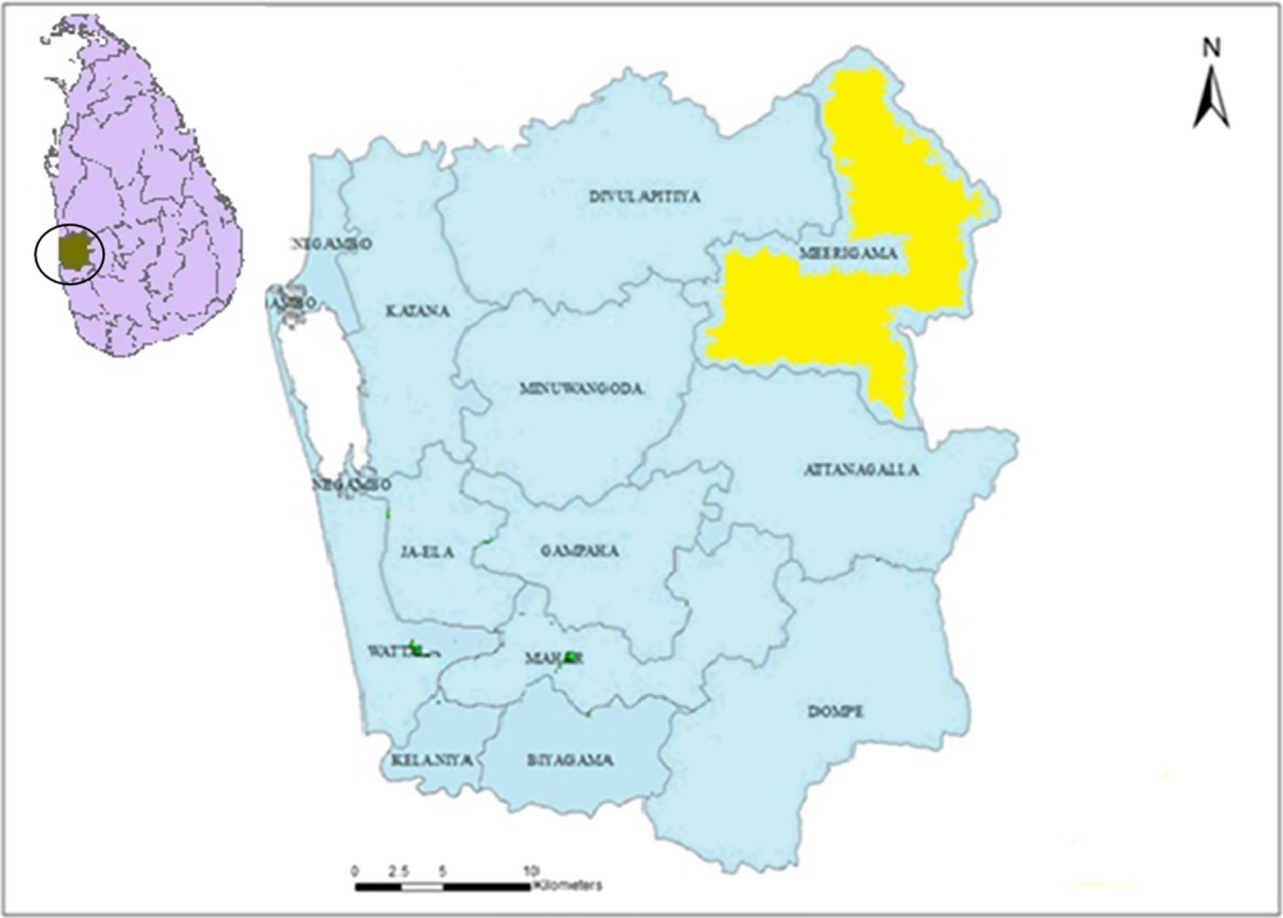
A map of the District of Gampaha showing the Divisional Secretariat of Mirigama. The district of Gampaha is located within the *circle* of the map of Sri Lanka positioned in the *left upper corner* of the figure. In the map of Gampaha, the Divisional Secretariat Mirigama is indicated in *yellow*

A study was conducted from May 2012 to February 2013 in the Divisional Secretariat of Mirigama within the District of Gampaha. In 2011, the Epidemiology Unit of Sri Lanka reported an annual incidence of 7.96 per 100,000 of the population for leptospirosis in the Divisional Secretariat of Mirigama, which, therefore, was selected as the site for this study [13]. Gampaha is located in the Western Province of Sri Lanka, in the Wet Zone of the country (Fig. 1). The annual mean temperature of the district ranges between 26.5 and 28.5 °C and the average annual rain fall is approximately 5000 mm [14]. It contains many small rivers, canals and spring heads, thereby presenting a favorable environment for the survival of leptospiral bacteria. The main occupation of the population is agriculture, especially rice farming involving the intensive use of water buffaloes and cattle.

### Collection of rodent blood samples and kidney tissue samples

Rodents were trapped in household areas, rice fields and paddy stores in the study area. Traps with baits were left overnight after obtaining written permission from the owner of the property, and rodents transported in traps to the Molecular Medicine Unit, Faculty of Medicine, University of Kelaniya, the following morning. Cardiac puncture was carried out to collect 1–2 ml of blood after first anesthetizing the rodents using diethyl ether, given according to the weight of the rodent [15]. A needle attached to a 5 cm^3^ syringe was inserted through the ventral abdominal wall just lateral to the xiphoid process, at an angel of 15°–20° above the plane of the abdomen and directed toward the heart, and cardiac blood collected before the heart stopped beating. The blood was immediately placed into a tube containing ethylenediaminetetraacetic acid (EDTA) held at 4 °C. The abdominal cavity was opened, the kidneys removed aseptically to a sterile vial, and stored at −80 °C until DNA extraction and PCR testing.

### Collection of urine samples from cattle

Mid-stream urine samples (approximately 50 ml) were collected from randomly selected cattle and buffaloes, into sterile plastic vials and transported to the laboratory at 4 °C. Samples were immediately centrifuged at ~5000×*g* for 15 min, the pellets were resuspended in 1 ml of 1 × phosphate buffered saline (PBS) [137 mM NaCl, 2.7 mM KCl, 4.3 mM Na_2_HPO_4_, 1.4 mM KH_2_PO_4_ (pH 7)], transferred into a 1.5 ml microcentrifuge tube and centrifuged at ~8000×*g* for 5 min. The supernatant was discarded and pellets resuspended in 1× PBS and stored at −20.0 °C until used for testing [16].

### DNA extraction

Deoxyribonucleic acid (DNA) was extracted from the kidney tissues and urine samples using the QIAamp DNA Mini Kit (QIAGEN) and QIAamp viral ribonucleic acid (RNA) Mini Kit (QIAGEN), respectively, according to the manufacturer’s instructions. DNA was stored at −20 °C until tested by PCR.

### Real time polymerase chain reaction

For DNA amplification, a real-time polymerase chain reaction (PCR) using primers 5′-GCG ATT CAG TTT AAT CCT GC-3′ and 5′-GAG TTA GAG CTC AAA TCT AAG-3′, targeting the *secY* gene of pathogenic *Leptospira*, was performed [17]. The assay was optimized using reference samples (*L. interrogans* icterohaemorrhagiae strain RGA) obtained from a reference laboratory (WHO/FAO Collaborating Centre on Leptospirosis, Meibergdreef 39, Amsterdam, Netherlands). The ability of the specific pair of primers to amplify the *secY* gene of pathogenic *Leptospira* strains was tested using the reference strain (*L. interrogans* serovar icterohaemorrhagiae strain RGA) alongside DNA and cDNA of pathogenic microorganisms, clinically important in the country (dengue, chikungunya, hantaviral infection, leshimaniasis, malaria, hepatitis and tuberculosis). DNA of the *Leptospira* saprophytic species, *L. biflexa* serovar patoc strain patoc 1, was also included in this test to determine assay specificity.

Polymerase chain reaction amplification was carried out in a total volume of 25 µl 1× Maxima^®^ SYBR Green-I/ROX qPCR master mix (Thermo Scientific) containing Maxima^®^ Hot Start *Taq* DNA polymerase and deoxynucleotide triphosphates (dNTPs) in an optimized PCR buffer made up to a final concentration of 2.5 mM MgCl_2_. Forward and reverse primers were added to a final concentration of 0.4 µM each followed by addition of 10 µl of extracted DNA (sample). Each PCR run included negative controls in which 10 µl of nuclease-free distilled water was added instead of template DNA. *L. interrogans* serovar Icterohaemorrhagiae, strain RGA, DNA (≤100 ng, 10 µl) was used as the positive control.

Polymerase chain reaction was carried out using Swift Spectrum 48 real time thermal cycler (Esco Healthcare Pvt Ltd, Singapore): initial denaturation at 95 °C for 10 min followed by 40 cycles of denaturation at 95 °C for 15 s, annealing at 54 °C for 30 s, extension at 72 °C for 30 s, and incubation at 72 °C for 7 min. melting temperature (Tm) analysis (70–94 °C with readings every 0.5 vC) was performed after a cooling step of 30 °C for 1 min according to the manufacturer’s instructions. The cut-off was set at threshold cycle (Ct) 35 which in our hands was the last cycle completely devoid of background noise. All experiments were repeated at least twice to test for reproducibility.

### Microscopic agglutination test (MAT)

Serum samples of rodents were separated from the rodent’s blood samples and sent to the Medical Research Institute (MRI), Colombo, Sri Lanka for testing by the microscopic agglutination test (MAT). MAT was performed with the non-pathogenic *L. biflexa* serovar Patoc, strain Patoc 1 setting a reciprocal titre of 100 as a positive result for identifying current and past infections with *Leptospira*. A titer between 20 and 100 was considered as indeterminate. Due to the unavailability of serovar-specific MAT (Standard-MAT) at the MRI, Sri Lanka, another study was carried out to compare the Patoc-MAT with standard MAT using patient serum samples. An acceptable level of agreement between patoc-MAT and standard-MAT was observed and therefore patoc-MAT was subsequently used [18].

### Statistical analysis

A Chi square test (Minitab 15 statistical software) was used for comparison of the categorical variable (male and female reservoir animals) at a 95% confidence interval; a p value <0.05 was considered to be significant.

## Results and discussion

### Rodents

Thirty eight rodents were captured from household areas (n = 18), paddy fields (n = 16), paddy stores (n = 3) and one commercial establishment (n = 1). Of the 38 rodents, 26 were female and 12 were male (Table 1). Detailed speciation of the rodents was not performed but global inspection indicated all rodents to be *Rattus rattus*. Four rodents (11%) were positive for *Leptospira* by PCR. Unfortunately, the PCR products were not suitable for subsequent sequencing for species identification because SYBR green interferes with the sequencing reaction and furthermore insufficient PCR product was produced for gel purification or column-based purification. Three samples (8%) were positive by MAT. The only positive MAT outcome with a reciprocal titer of 100 coincided with a positive PCR result. No indeterminate titers were found. The prevalence of leptospiral carriage was 11% (3/26) and 8% (1/12) in female and male rodents, respectively; this difference was not significant (Table 1).

**Table 1:** Laboratory test results of reservoir animals by gender

Type of animal	Gender	Laboratory test results			P value
		Positive	Negative	Total	
Rodents	Female	3	23	26	0.765
	Male	1	11	12	
	Total	4	34	38	
Cattle/buffalos	Female	2	35	37	0.068
	Male	3	10	13	
	Total	5	45	50	

These results indicate that rodents represent a reservoir of *Leptospira* in the Division of Mirigama. It should be noted that DNA extracted from kidney tissues might contain inhibitors of the PCR [17], and so the PCR results may underestimate the true prevalence, and the carrier rate of 11% may be a pessimistic estimate. As the rodents were caught at or near domestic premises and farms, these present a high exposure risk for humans. Considering the similar ecological conditions found outside the District of Gampaha, we assume that this observation is also true outside the division. Our finding is consistent with several other reports that rodents present a reservoir host in most parts of the world [9, 11, 15, 19, 20].

The value of MAT for the identification of carriers is disputable. Many, if not most natural hosts do not produce antibodies against the *Leptospira* serovar(s) they are carrying [21] and hence, MAT may underestimate the carrier rate. The MAT performed in this study has an additional drawback, in that MRI, the primary and only site in Sri Lanka performing MAT, is using serovar Patoc only. MAT is usually executed with a panel of serovars to achieve a certain level of serovar specificity, particularly when analyzing convalescent serum samples. The saprophytic serovar Patoc is often included to detect cross-agglutinating antibodies in samples collected from mainly acute patients. However, its ability to detect cross-reacting antibodies from past infections is limited. Moreover, even a low titre found in a MAT test performed with a suitable panel on serum samples from animals can be a meaningful indication of infection [22]. However, a low titre of Patoc is difficult to interpret and could indicate either persistent infection with a recent re-infection with a homologous serovar or a recent infection with a heterologous serovar. Whereas MAT titers with Patoc are difficult to interpret in the case of an individual animal, our finding of a measurable titre in 8% of the rodents with Patoc is consistent with a considerable infection rate and hence supports our PCR observation that rodents represent an infection source in the study area.

### Bovines

Fifty urine samples were collected from 45 cattle and 5 buffaloes, selected randomly. Of the 45 cattle, 35 were females and 2 buffaloes were females. In total, five samples (10%) were positive by real-time PCR. The prevalence of leptospiral carriage was 9% (4/45) and 20% (1/5) in cattle and buffaloes, respectively. Of the four positive cattle, 3 (75%) were male. The one positive buffalo was female. The prevalence of leptospiral infection was 40% (2/5) and 7% (3/45) in female and male bovines, respectively, not a significant difference (Table 1).

Furthermore, for these bovines, we argue that our PCR data represent an underestimation of prevalence for the following reasons. Animals display intermittent shedding of leptospires and several consecutive samples should be collected to establish whether an individual animal is a carrier [19, 22], but for practical reasons we collected only a single sample. For DNA extraction, bacteria were collected by an initial centrifugation of urine samples at ~5000×*g*. It is conceivable that at this relatively low speed the leptospires were not all pelleted and hence the number in the final samples was below the lower PCR detection limit, and false negatives are likely to have been recorded. The extent of any underestimation remains unknown, but the true carriage rate may exceed the average 10% we found in cattle and buffaloes. Thus we conclude that bovines may present a public health risk in the Divisional Secretariat of Mirigama, Gampaha, which reports to the Epidemiology unit in Sri Lanka, in concurrence with the high number of local human leptospirosis cases recorded. This is in accordance with a recent study by Gamage and coworkers [23] who concluded that cattle act as a reservoir animal for a variety of pathogenic *Leptospira* spp. in Sri Lanka.

## Conclusions

Based on our data and those of others [1, 6, 20, 23], we believe that rodents and bovines, living in close vicinity of humans, form two infection reservoirs in the Divisional Secretariat of Mirigama and beyond. The identification of the same serovar in both human patients and in suspected reservoir hosts would be needed for direct evidence, and further research will focus on isolating leptospires from patient and animals enabling detailed typing of the infecting serovars, and the identification of other potential reservoir animals.

## Abbreviations

PCR: polymerase chain reaction
MAT: microscopic agglutination test
EDTA: ethylenediaminetetraacetic acid
PBS: phosphate buffered saline
DNA: deoxyribonucleic acid
RNA: ribonucleic acid
dNTPs: deoxynucleotide triphosphates
Tm: melting temperature
Ct: threshold cycle
MRI: Medical Research Institute
